# Impact of Silicon Addition on the Development of Gelled Pork Lard Emulsions with Controlled Lipid Digestibility for Application as Fat Replacers

**DOI:** 10.3390/gels9090728

**Published:** 2023-09-07

**Authors:** Susana Cofrades, Marina Hernández-Martín, Alba Garcimartín, Arancha Saiz, M. Elvira López-Oliva, Juana Benedí, María Dolores Álvarez

**Affiliations:** 1 Institute of Food Science, Technology and Nutrition (ICTAN-CSIC), 28040 Madrid, Spain; a.saiz@ictan.csic.es; 2 Physiology Department, Pharmacy School, Complutense University of Madrid, 28040 Madrid, Spain; marinh04@ucm.es (M.H.-M.); elopez@farm.ucm.es (M.E.L.-O.); 3 Pharmacology, Pharmacognosy and Botany Department, Pharmacy School, Complutense University of Madrid, 28040 Madrid, Spain; a.garcimartin@ucm.es (A.G.); jbenedi@ucm.es (J.B.)

**Keywords:** gelled emulsions, fat digestion, silicon, bioaccessibility, gel properties

## Abstract

Pork lard gelled emulsions stabilized with two proteins [soy protein concentrate (SPC) or a pork rind protein extract (PRP)], both with and without added silicon (Si) from diatomaceous earth powder, were gelled by microbial transglutaminase and к-carrageenan. These gelled emulsions (GEs), intended as fat replacers, were evaluated in different aspects, including microstructure and technological properties during chilling storage. In addition, in vitro gastrointestinal digestion (GID) with an analysis of lipolysis and lipid digestibility was also evaluated. All GEs showed adequate technological properties after 28 days of chilling storage, although the SPC-stabilized GEs showed better gravitational and thermal stability (~4% and ~6%, respectively) during chilling storage than the PRP-stabilized ones (~8 and ~12%, respectively). PRP developed larger flocculates restricting pancreatic lipase-mediated lipolysis during intestinal digestion. The addition of Si to both GE structures protected them against disruption during in vitro digestion. Accordingly, Si appears to slow down fat digestion, as reflected by higher triacylglycerides content after GID (15 and 22% vs. 10 and 18% in GEs without Si) and could become a potential candidate for use in the development of healthier meat products.

## 1. Introduction

Oil-in-water (O/W) emulsions elaborated with unsaturated liquid oils (vegetable and marine origin) have been widely used in the food industry as saturated fat replacers and as a vehicle for different hydrophilic or lipophilic bioactive compounds. This is a strategy employed to produce healthier meat products [1]. However, these systems are thermodynamically unstable because of the positive free energy generated by interfacial tension, and the oil and water phases tend to separate over time due to certain processes. Therefore, their incorporation in the reformulation of healthier meat products may negatively influence their stability and acceptability when compared with animal-fat meat products. On the other hand, the emulsification process can favor the action of digestive lipases by simplifying the emulsification that occurs within the gastrointestinal tract, thus increasing the O/W interfacial area for lipase adsorption [2,3]. In this sense, different authors reported that the release of free fatty acids (FFA) increases when lipids are administered in smaller emulsified systems [4,5]. In this regard, emulsion stability can be greatly improved by entrapping oil droplets within a solid-like three-dimensional aqueous network (hydrogels). Protein-stabilized emulsions can be converted into gelled emulsions (GE) through several food processing operations, including microbial transglutaminase (MTG) treatment, through covalent crosslinking between protein molecules adsorbed on different droplets [6]. The enzyme MTG catalyzes acyl transfer reactions between intra- or interchain protein lysine and glutamine peptide residues [7]. Due to the covalent character of the crosslinks, MTG-induced protein gels exhibit elastic behavior and are heat stable, making these GEs suitable for reformulating heat-treated meat products [1], as they provide stability and texture. On the other hand, these formed structures could limit fat digestibility, which is a desirable feature in low-caloric diets and could help in the management of important chronic metabolic diseases such as obesity and type 2 diabetes mellitus (T2DM) that are associated in our current society with hypercaloric diets. In this context, Gayoso et al. [8] reported that the structural and physical state of gelled double or simple emulsions results in a slower triacylglyceride (TAG) hydrolysis in comparison with that of the ungelated simple and double control emulsions.

Soy proteins have been traditionally used to stabilize food emulsions due to their suitable functional properties [9]. However, in the quest to identify novel protein sources from the by-products of the food industry, some collagen-rich protein ingredients extracted from pork and bovine rinds and hides have barely been studied, despite their potential use as emulsifiers and gelling agents in the food industry [10]. The benefits of functional pork collagen proteins (PRP) include improved water binding, better texture, reduced syneresis, improved emulsifying capacity, and enhanced organoleptic characteristics, providing a new technological strategy for developing functional low-fat meat products [11].

The enrichment of foods with bioactive compounds is an increasingly important area in the food industry. In this context, silicon (Si) is an essential micronutrient with significant beneficial health effects, as it is a neuroprotector and a bone mineralization inducer, and presents other less-known functions related to hypoglycemic and hypolipemic properties, as well as antiapoptotic and antioxidant capacities [12,13]. Additionally, Si has been previously added to a meat model system obtaining important benefits in the treatment of metabolic syndrome in adult rats, modulating the duodenal cholesterol absorption [14] and improving the lipoprotein profile and liver function [12,13]. However, it must be taken into account that Si bioavailability depends on the matrices in which it is included, and this may affect its efficacy [15]. In a previous study, a “synthetic” preparation of organic Si was incorporated into the aqueous phase of O/W soft-like emulsions stabilized with soy protein concentrate (SPC) and with methylcellulose (MC), which was added sequentially to the emulsion [16]. The results showed that the presence of Si produced a slight reduction in the release of FFA during in vitro gastrointestinal digestion (GID) of the SPC-stabilized emulsions; however, this effect was lost when MC was also incorporated. In the search for alternative sources of Si, a food supplement rich in organic and natural Si was found: diatomaceous earth powder (DP). DP is composed of millions of hard vegetable shells from unicellular algae called diatoms. When consuming this Si present in DP, a large part of it upon contact with the gastric juices of the organism is transformed into orthosilicic acid, which is the most bioavailable form of Si for humans [17,18]. Despite the great potential functional effects of Si, there are very few studies on its use in emulsions as a delivery system for bioactive compounds, and even fewer studies on GEs and how gel structure and digestion behavior affect its bioaccessibility. DP is easier to handle than synthetic organic Si and shows high solubility not only in water but also in oil, making it interesting to evaluate its potential limiting effect on lipid digestibility, as well as its bioaccessibility when incorporated into the oil phase of a GE.

To date, GEs have been prepared with different healthy oils, either to obtain ingredients with a consistency similar to that of animal fat or to control lipid digestibility [8]. However, there are no studies on structured emulsions made with pork lard and containing a bioactive compound such as Si. Therefore, the aim of this study was to develop GEs elaborated with animal fat that was stabilized by two different emulsifying proteins (SPC and PRP) and with and without added Si, evaluating aspects related to their physicochemical properties, structure, and stability during chilling storage and in vitro lipid digestibility.

## 2. Results and Discussion

The gel-like structures were obtained from emulsions stabilized with SPC and PRP, using the enzyme MTG through a cold gelling process. к-carrageenan was also added since it is well known that different food proteins in combination with carrageenans present synergistic effects on texture properties/ and they have also been used to make different types of cold-set emulsion-filled gels. The aim was to create solid and robust systems with characteristics resembling animal fat, which is commonly used in the elaboration of meat products.

### 2.1. Macro- and Microstructure

**Figure 1:**
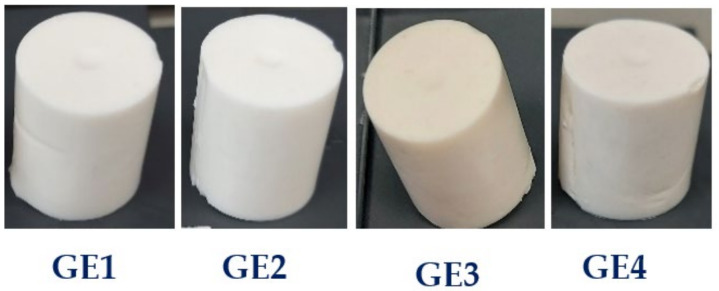
General appearance of the four gelled emulsions. GE, gelled emulsion; GE1, containing 3% soy protein concentrate (SPC), GE2, containing 3% pork rind protein extract (PRP); GE3, containing 3% SPC and 1.5% diatomaceous powder (DP) and GE4 containing 3% PRP and 1.5% DP. All four GEs contained 40% pork fat, 1% MTG, and 0.75% к-carrageenan.

**Figure 2:**
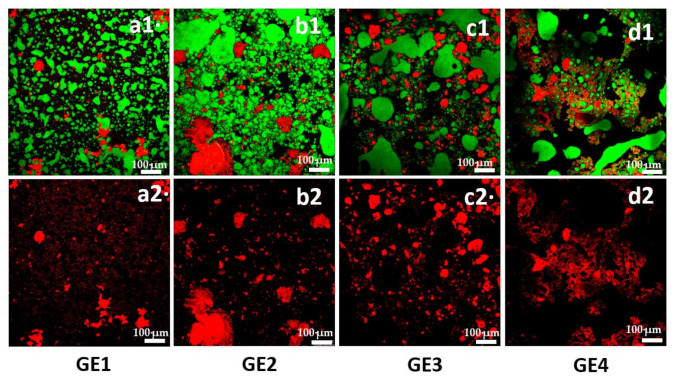
CLSM of the four GEs. The proteins and polysaccharide were stained with Fast Green (red), while the oil droplets were stained with Nile Red (green); (**a1**–**d1**) are the composite images of protein and the oil droplet, (**a2**–**d2**) are the images of protein in (**a1**–**d1**). Scale bar = 100 µm. For sample denomination, see Figure 1.

Representative images of the macro- and microstructure of the freshly prepared or non-digested GEs are shown in Figure 1 and Figure 2, respectively. Figure 1 shows that all GEs were self-supporting and exhibited a white color with a smooth surface, suggesting that the incorporation of MTG and к-carrageenan promoted the gelation process (Figure 1). In all GEs, a gel-like structure consisting of dense and packed droplets trapped within the crosslinked EP/MTG continuous phase was observed. In general, the images displayed a crosslinked network polymer (probably formed by the EP and MTG) (in red; Figure 2(a2)–(d2)) with entrapped emulsified oil droplets (in green; Figure 2(a1)–(d1)), as observed by Farjami and Madadlou [19]. However, the structures varied depending on the EP used and the presence or absence of DP as a source of Si.

In the SPC-stabilized GE (GE1), the oil droplets were smaller and more homogeneously distributed in the gel matrix, showing a more uniform network structure than when PRP was used (GE2) (Figure 2(a1,b1). This can also be seen in Figure 2(a2) where the continuous network exhibits smaller and more uniformly sized pores or holes than in Figure 2(b2), in which the network formed by the PRP and the MTG is more discontinuous, with larger and more numerous aggregates. Moreover, it is possible to observe some larger droplets in PRP emulsions (GE2, Figure 2(b1), indicating that PRP molecules may have thickened and gelled the water droplets, which led to larger droplet sizes in the overall system. This latter structure can result in a lower ability to stabilize the emulsion (Figure 2(b1)). This behavior could be attributed to various factors. On the one hand, to the different structures of the EP studied, SPC is a globular protein with exceptional functional properties [9], while PRP has a high proportion of collagen and a fibrillar structure. Although PRP exhibits good water- and fat-binding properties, as well as texturizing properties, its emulsifying capacity appears to be lower than that of SPC. In this sense, Surh, Decker, and McClements [20] pointed out that fish gelatin showed less emulsifying activity than globular proteins due to its relatively low surface activity. On the other hand, the different structure of the EP studied is also responsible for the disparate affinity of MTG and both proteins. In this regard, it has been stated that casein, sodium caseinate, gelatin, myosin, 7 S globulin, and 11 S soy are good substrates for MTG [21]. In contrast, proteins such as collagen are also reactive to MTG but to a lesser degree than the aforementioned substrates [22,23]. In turn, the addition of Si also modified the structure of the GEs (Figure 2(c1,d1,c2,d2). The addition of Si to the oil phase may have interfered with the EP’s ability adequately to emulsify the oil droplets. This suggests that the structure of the continuous phase of the GEs stabilized with SPC and PRP in the presence of Si presents a more compact and aggregated structure with stronger crosslinking compared with the absence of Si, where the fat droplets are more strongly trapped in the matrix (Figure 2(c1,d1) vs. Figure 2(a1,b1). This behavior was even more evident when PRP was used as the EP. Cofrades et al. [16] reported that the incorporation of Si in biopolymeric emulsions stabilized with SPC and methylcellulose resulted in a decrease in the population of smaller droplets while favoring the presence of larger ones.

### 2.2. Technological Properties during Chilling Storage

Emulsion properties such as color, texture, and water and fat binding capacity are attributes that can significantly influence consumer purchasing decisions. Therefore, they are crucial properties to consider during the development of fat substitutes and meat products. Moreover, these characteristics may also be affected by chilling storage, given the nature of either of these newly formulated systems or the products to which they will be added.

#### 2.2.1. Instrumental Texture and Color

**Figure 3:**
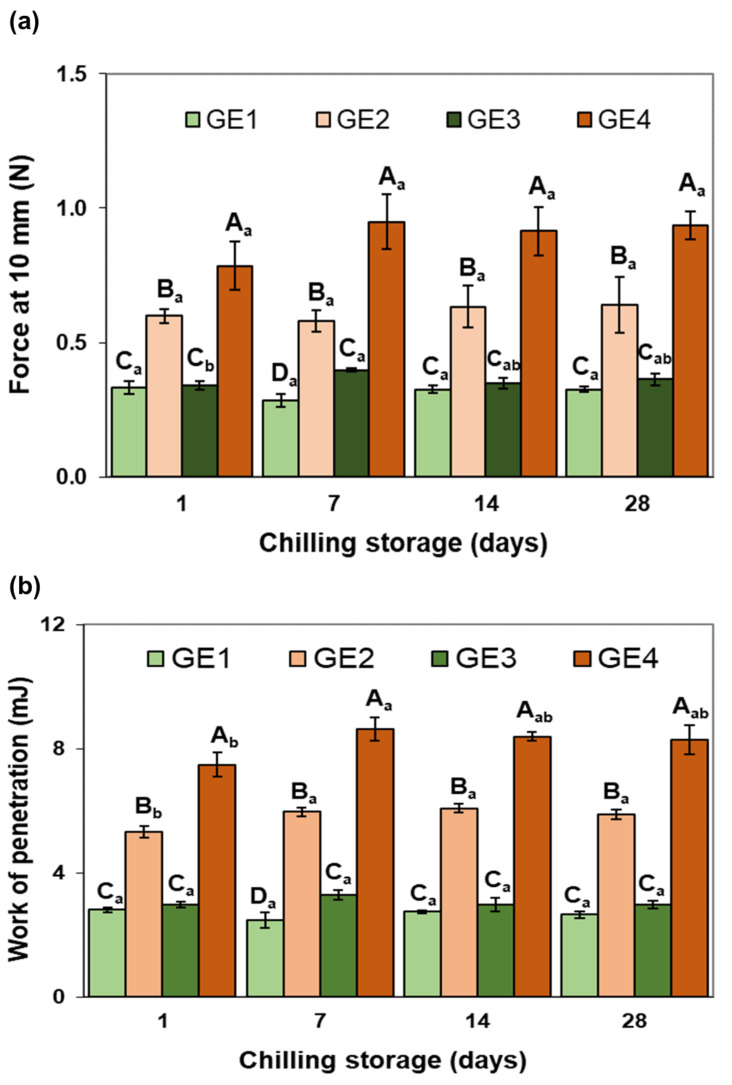
Effects of GE composition (emulsifying protein (EP) and silicon (Si) presence) and chilling storage on textural parameters from a penetration test. Each value is the mean of 4 replicates and vertical bars are standard errors. (**a**) is force of penetration at 10 mm; (**b**) is work of penetration. ^A–D^ Effect of GE composition. For each textural parameter and for the same chilling storage time, mean values without the same letter are significantly different (*p* < 0.05). _a,b_ Effect of chilling storage. For each textural parameter and for the same GE composition, mean values without the same letter are significantly different (*p* < 0.05). GE, gelled emulsion. For sample denomination, see Figure 1.

Regarding the textural parameters of the GEs (Figure 3), in fresh samples (1 day of chilling storage), the EP (SPC vs. PRP) had a significant effect on the force at 10 mm (Figure 3a) and work of penetration values (Figure 3b). These were significantly higher in both GEs prepared with PRP (GE2 and GE4) than those containing SPC (GE1 and GE3). Therefore, it can be stated that the use of PRP increases the structural strength of the GE in comparison with SPC. This behavior can be attributed, as previously mentioned, to the different structure and composition of SPC and PRP, which originated a compact and more discontinuous structure with larger aggregates in the PRP-stabilized GEs (Figure 2(b1)). In turn, the addition of Si only showed a significant effect in the GEs formulated with PRP, resulting in higher values for both textural parameters compared with its Si-free counterpart. The texture results seem to be consistent with the microstructure images of the non-digested GEs, where it is possible to appreciate that the PRP-stabilized GE (GE4) exhibited a more compact and denser network than the one formed by SPC with Si (GE3) (Figure 2(c1,d1)). Likewise, GE4 had more irregular and less spherical fat globules than GE2 (Figure 2(b1,d1)).

Chilling storage had a minimal effect on the texture of the GEs so at the end of the storage period, the values of both parameters were similar to those of freshly made GEs. This would reflect the high structural stability of all GEs over time. In terms of GE color, all fresh GEs had a similar whitish solid-like appearance at room temperature, resembling that of pork lard (Figure 1). However, GE color parameters were slightly affected by the formulation (Table 1). GE3, stabilized with SPC and with Si added to the oil phase, exhibited lower (*p* < 0.05) lightness and higher redness and yellowness than the rest of the GEs. This may be attributed to the interactions between Si, the melted pork lard, and SPC. In addition, GE color was hardly affected by storage (Table 1). The lightness and yellowness of all GEs remained unchanged during storage, whereas the redness values decreased in all GEs at the end of the storage period. In general, these changes have a minimal impact and, therefore, do not compromise the use of these GEs as fat replacers.

#### 2.2.2. Gravitational and Thermal Stability

**Figure 4:**
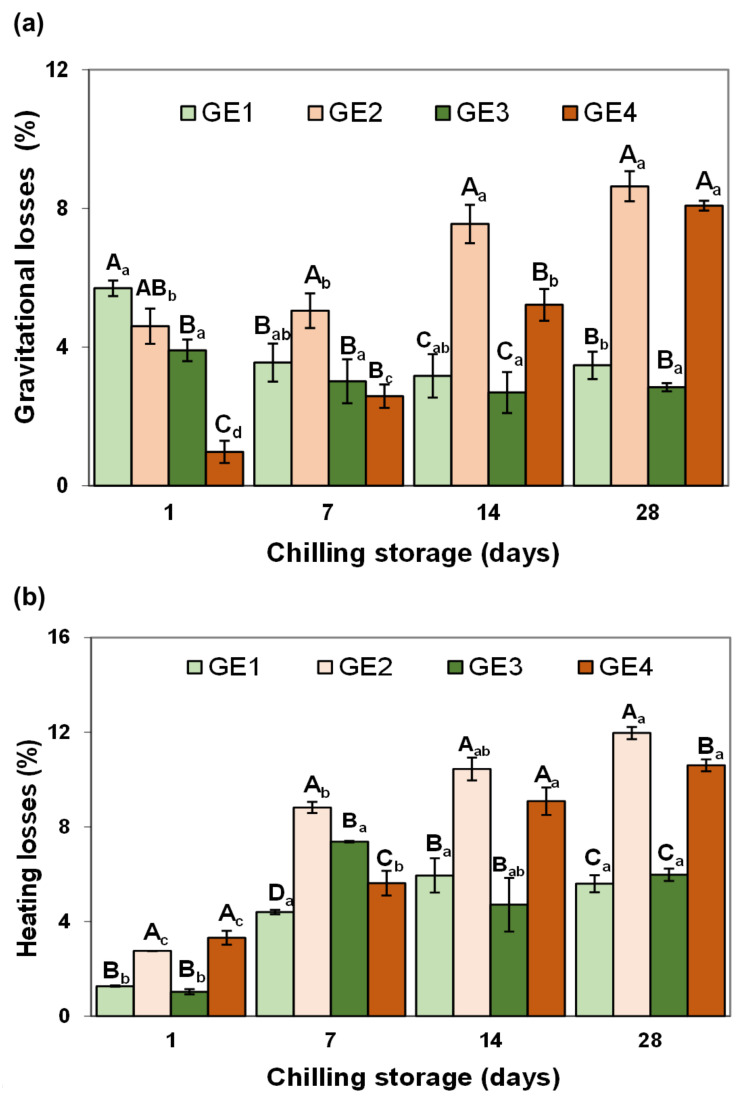
Effects of GE composition (emulsifying protein (EP) and silicon (Si) presence) and chilling storage on gravitational and heating losses. Each value is the mean of 4 replicates and vertical bars are standard errors. (**a**) is gravitational losses; (**b**) is heating losses. ^A–D^ Effect of GE composition. For each stability measurement and for the same chilling storage time, mean values without the same letter are significantly different (*p* < 0.05). _a–c_ Effect of chilling storage. For each stability measurement and for the same GE composition, mean values without the same letter are significantly different (*p* < 0.05). GE, gelled emulsion. For sample denomination, see Figure 1.

The stability of the GEs is related to their ability to withstand external changes over time or heating, which depends on their composition [24]. The greater the stability of the GE, the less likely its properties are to change. In the context of meat product reformulation, GE stability is a technological property that holds paramount importance as most meat products undergo some type of storage and/or heating process, and consequently, it must be considered. Figure 4 displays data concerning the change in emulsion stability of the GEs measured by gravitational (Figure 4a) and heating losses (Figure 4b) during chilling storage.

As regards the gravitational stability of the GEs, in freshly prepared samples without Si, (GE1 and GE2), the EP used to stabilize the emulsions did not have a significant effect on the exudate released, with losses ranging between 5.7 and 4.6% for GE1 and GE2, respectively (Figure 4a). However, the addition of Si to both GEs overcame the observed initial losses, reaching practically insignificant loss values (<1%) in the case of GE4. During chilling storage, the opposite effect was observed, noting that the PRP-stabilized GE (GE2) significantly increased gravitational losses after 14 days of storage, observing losses of approximately 8% at the end of the storage. However, the addition of Si counteracted these losses for up to 14 days of storage, noting that at the end of the storage time, Si loses its effect (Figure 4a). On the contrary, the SPC-stabilized emulsions (GE1 and GE3) presented greater stability during conservation, resulting in final losses of less than 4%. Therefore, the addition of Si had a greater impact on the gravitational loss than the EP in the non-digested GEs, although this effect was lost during storage time as the effect of the EP prevailed. 

Concerning the thermal stability of the emulsions (heating losses), the SPC-stabilized GE (GE1) initially presented favorable fat- and water-binding properties, showing low liquid release (<2%) during heating (Figure 4b), while GE2, prepared with PRP, showed higher and significant heating losses, although still relatively low. The presence of Si had no effect on any of the GEs. In general, heating losses increased during chilling storage in all the GEs, finding significantly higher values (*p* < 0.05) in GE2, with PRP, compared with GE1, stabilized with SPC. At the end of the storage period, GE1 and GE3 showed similar and lower losses than GE2, respectively, noting a stabilizing effect of Si on this latter GE (GE4 vs. GE2 at 28 days, Figure 4b). Therefore, the EP had more influence on thermal stability than the presence of Si, similar to the findings for gravitational stability. Although these results are difficult to interpret, it is possible to attribute the observed effects to the different formulations and, thus, to the proteins used to stabilize the GEs as well as to the presence of Si and the interactions between the different food components during storage. Overall, the emulsion stability values, fall within the expected ranges found in various studies on GEs [1]. The greater stability observed in the GEs containing SPC as the EP (GE1 and GE3), compared with those formulated with PRP (GE2 and GE4), could be due to the good interfacial and emulsification activities shown by vegetable proteins in comparison with collagen [25]. Taking into account that the main protein present in the pork rind protein extract (PRP) is collagen, these results would confirm the worse emulsifying capacity of PRP compared to SPC. On the other hand, soy protein is a better substrate for MTG than collagen from pork rind and bovine hides [21], which may also contribute to the observed differences. These results are consistent with the textural parameter values and microstructure images observed in these GEs, where a more heterogeneous structure was observed in the GEs stabilized with PRP, presenting larger oil droplets in comparison with SPC (Figure 2(a1,b1)). In this sense, other studies have demonstrated that emulsions containing larger droplets exhibit greater oil destabilization than those with smaller ones [20]. Although the particle size of the GEs was not determined, the droplet size of the liquid-like emulsions (primary emulsions) made with SPC and PRP was registered as the volume-mean diameter D[4,3]. As expected, SPC resulted in smaller oil droplet sizes (~50 µm), whereas with PRP, these were larger (~112 µm), confirming our results in GEs.

#### 2.2.3. Lipid Oxidation

**Figure 5:**
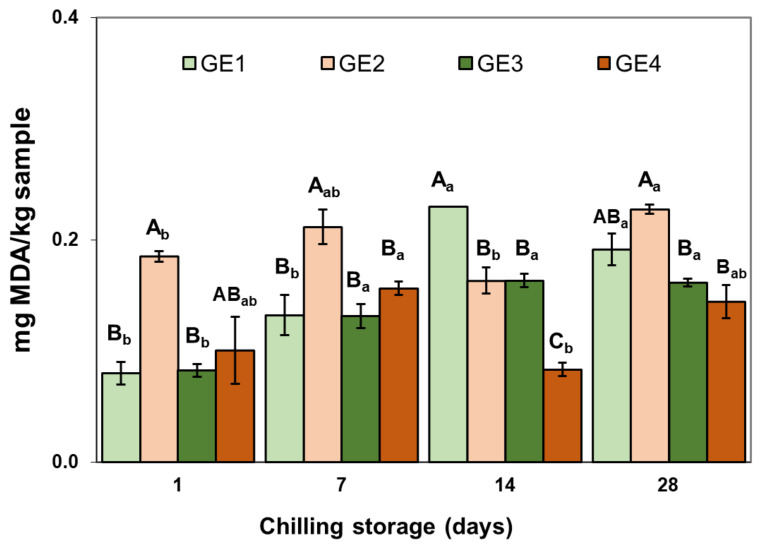
Effects of GE composition (emulsifying protein (EP) and silicon (Si) presence) and chilling storage on lipid oxidation (TBARS). Each value is the mean of 3 replicates and vertical bars are standard errors. ^A–C^ Effect of GE composition. For the same chilling storage time, mean values without the same letter are significantly different (*p* < 0.05). _a,b_ Effect of chilling storage. For the same GE composition, mean values without the same letter are significantly different (*p* < 0.05). GE, gelled emulsion. For sample denomination, see Figure 1.

Thiobarbituric acid reactive substances (TBARS) were influenced by the formulation (both EP and presence or absence of Si) and chilling storage time (Figure 5). Initially, all the samples presented relatively low TBARS values (0.08–0.19 mg MDA/kg of sample), similar to those found in other GEs [1,26]. The GEs exhibiting the highest oxidation level were the ones that contained PRP as the EP, without Si (GE2). These low initial values of lipid oxidation could be associated with the fact that the GEs were made with animal fat, rich in saturated fatty acids (SFA) and monounsaturated fatty acids (MUFA), which are less prone to lipid oxidation than vegetable fats with a higher content of polyunsaturated fatty acids (PUFA). Additionally, it is noteworthy that the gel formation was enzymatic using MTG and cold, and not carried out by heat treatment. The presence of Si in the PRP-stabilized GE (GE4) showed lower TBARS values compared with the absence of Si (GE2), whereas there was no effect on the SPC-stabilized GE (GE1), which may be due to the low initial oxidation level observed in this sample. Irrespective of the composition, the changes in TBARS contents over the storage period of the GE, although significant, were not relevant because the values did not exceed 0.2 mg MDA/kg at the end of the storage period (Figure 5). Therefore, these systems can be considered to have low susceptibility to oxidation even after 30 days from preparation.

### 2.3. Extent of Lipolysis during In Vitro Digestion

The impact of the EP (SPC vs. PRP) and the presence or not of Si from DP as a functional ingredient in the GEs, all structured with MTG and к-carrageenan, on the extent of lipolysis was evaluated by high-performance size-exclusion liquid chromatography (HPSEC). The composition of the digestion products, including the loss of TAG and the formation of hydrolytic compounds (diacylglycerides (DAG), monoacylglycerides (MAG), and FFA)) at the end of 90 min of intestinal digestion (ID–90), allowed for the determination of the expected reduction in lipid digestion. The fat composition of the four digested GEs at ID–90 is shown in Table 2. Initially, the lipid composition of the GEs was typical of pork lard, with 99.9% TAG, and there were no differences among samples.

Regarding the effect of EP on the degree of lipolysis, the fat extracted from the PRP-stabilized GE (GE2) at the end of ID, revealed a significantly (*p* < 0.05) higher TAG content, and lower FFA content, in the absorbable fraction (MAG + FFA) and digestibility compared with the SPC-stabilized GE (GE1). This result is according to the consistency of the GEs, in the sense that the softer gels (GE1 and GE3) could be degraded much faster than the harder GEs (GE2 and GE4) during in vitro GID. Other authors have explained that this could be due to the fact that harder gels have a more compact particulate gel structure and there is more crosslinking between the EP within the gel [27].

The presence of Si in both GEs with Si resulted in a significantly higher TAG content and lower FFA content and degree of digestibility with respect to the GEs without Si. These results suggest that using PRP as the EP leads to a lower degree of digestion compared with SPC and that the presence of Si in both GEs also reduces fat digestion at the end of ID (ID–90). As for Si, the reduction of fat digestibility could be expected from previous results; Garcimartín et al. [12] observed that the inclusion of Si in a meat matrix allowed a notable reduction of lipemia in a metabolic syndrome rat model. In turn, Garcimartín et al. [12] demonstrated the hypotriglyceridemic effect of Si from the first administration. Recently, our research team found that the presence of Si in an SPC-stabilized emulsion reduced fat digestibility after 90 min of ID with respect to the emulsion without Si. This behavior could be attributed to the fact that the continuous phase structure of the GEs stabilized with SPC and PRP, and in the presence of Si, presented a more aggregated and compact structure with stronger crosslinking than in the absence of Si, where the fat droplets were strongly trapped inside (Figure 2(c1,d1) vs. Figure 2(a1,b1)). This could limit the access of bile salts and pancreatic lipase to the surface of the oil droplets and, therefore, reduce digestibility. Similar results were reported by Fontes-Candia et al. [28] in a GE with curcumin incorporated in the oil phase. These authors reported that the presence of curcumin limited the formation of the mixed bile salt-digestion products lamellae/micelles, and induced the formation of a greater number of larger vesicles, curcumin being most likely located within the interior of these structures.

### 2.4. Confocal Laser Scanning Microscopy of the Digested GE

**Figure 6:**
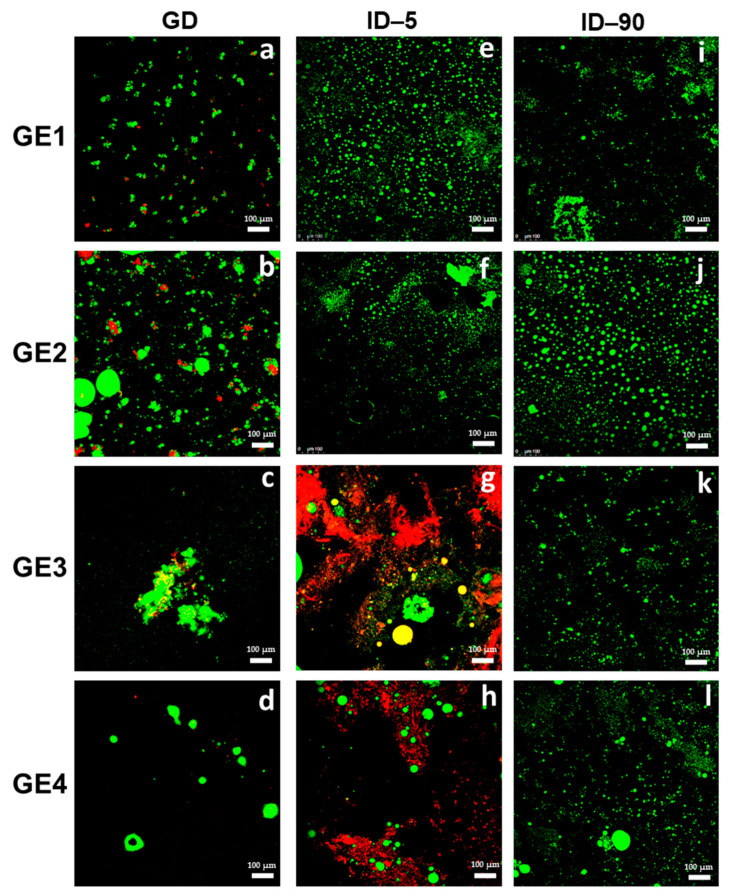
CLSM of the four GEs throughout the different in vitro gastrointestinal digestion (GID) phases. (**a**–**d**) are the images after GD of each GE; (**e**–**h**) are de images after ID-5 of each GE; (**i**–**l**) are the images after ID–90 of each GE. Scale bar = 100 µm. GD, gastric digest; ID–5; intestinal digest at min 5; ID–90, intestinal digest at min 90; GE, gelled emulsion. For sample denomination, see Figure 1.

The structural changes of the GEs during in vitro GID were also monitored using CLSM. In vitro GID generated changes in the microstructure of all the non-digested GEs (Figure 2), and the microstructure of their corresponding digests after the different in vitro GID phases (GD, ID–5, and ID–90) is shown in Figure 6.

In all gastric digests (GD) (Figure 6a–d), some fat droplets appeared flocculated due to pepsin digestion. These flocs appeared to be larger in the SPC-stabilized GEs. This implies that the proteolysis of the interfacial layer of fat droplets is greater in SPC than in PRP and, therefore, the thickness of the interfacial layer decreases, promoting the flocculation of oil droplets [29]. This could be a consequence, as previously discussed with other properties, of the structural difference between soy globular proteins, which contribute to the formation of more stable emulsions, and collagen, the main protein found in PRP, which produces fewer stable emulsions. In fact, stable emulsions under acidic conditions and emulsifiers causing a weak to moderate interfacial displacement of gastric lipase can result in significant degrees of lipolysis in the gastric compartment [30]. Furthermore, it is assumed that globular proteins are more easily accessible to digestive enzymes than fibrillar proteins. This observation is aligned with the gel texture or consistency, given that the softer gels seemed to be broken down into smaller particles than the harder gels, as stated by Luo et al. [27] in whey protein GEs. According to these authors, this could be due to hard gels having a more compact particulate gel structure and greater crosslinking between whey proteins within the gel [6]. Consequently, some cleavage sites may change and become less accessible for pepsin during GD, leading to slower hydrolysis of whey proteins. Even though whey proteins in both gels started to be hydrolyzed by pepsin at approximately the same time, the rate of proteolysis was slower in the hard gel (Figure 6b,d). The faster proteolysis and degradation of the soft gel may have led to the earlier and more severe occurrence of oil droplet coalescence in the gel particles from the soft gel (Figure 6a,c). A key factor contributing to the slower proteolysis and degradation of the hard gel could have been the slower diffusion rate of pepsin in its gel particles.

At min 5 of intestinal digestion (ID–5), a large number of small particles uniformly dispersed were observed in the digest from both gels without Si (GE1 and GE2), indicating the release of oil droplets from the protein matrix (Figure 6e,f). Conversely, a smaller number particles that were more heterogeneously distributed in the matrix was observed in the GEs with Si (Figure 6g,h).

A fact to highlight is the presence of the continuous phase network that still existed in significant amounts in the samples with Si (GE3 and GE4), which influenced the subsequent digestion process in the small intestine (Figure 6g,h). This fact could indicate that the presence of Si hinders or restricts access of digestive enzymes (pepsin and lipase) to the oil droplets. At the end of the ID (ID–90) (Figure 6i–l), and in all the GE digests, smaller oil droplets were observed than at ID–5, indicating digested oil droplets or the formation of mixed micelles and vesicles, and also some larger particles, which could have been undigested and/or coalesced/flocculated oil droplets.

There are no previous studies in which the effect of Si in emulsions on lipid digestibility in vitro has been evaluated. However, in a previous study by our group, it was found that the presence of Si in SPC-stabilized emulsions reduced lipolysis.

In summary, the results indicate that the digestion from the different GEs depends on the EP used to stabilize the GEs and the presence or absence of Si and that the behavior could be associated with the texture/consistency/structure of the freshly prepared or non-digested GE. It seems that the harder gels containing PRP (GE4 and GE2) were more resistant to degradation and digestion in the small intestine than those from the softer GEs with SPC (GE1 and GE3). Therefore, GE4 had the lowest fat digestibility and more gel-solid texture, despite presenting lower thermal stability and being somewhat less stable during chilling storage.

## 3. Conclusions

Solid gel-like structures from pork lard, MTG, and к-carrageenan with two different EP (SPC and PRP) and with Si added to the oil phase were prepared. The SPC-stabilized GEs with and without Si were softer and presented higher gravitational and thermal stability during chilling storage than the PRP-stabilized ones. The EP used had a more significant effect on the technological properties than the presence of Si in all the GEs. Chilling storage hardly affected these properties. When subjecting the GEs to an in vitro GID process, the type of structures generated because of the oil lipolysis depended on the protein type and the presence of Si. Regarding the protein type, the fibrillar protein present in PRP resulted in large flocculates that hindered the access of pancreatic lipase to the lipids within the droplets. In relation to Si, the structures of both GEs without added Si were easier to disrupt during the in vitro digestion process than the ones with Si, which presented remains of the initial matrix during the intestinal phase. Therefore, the presence of Si seems to limit the formation of digestion products and, consequently, fat digestion. These results reflect the importance of the GE composition in the different types of digestion products formed during in vitro GID, which would determine the intestinal transport and absorption of the produced compounds. These GEs could be used as healthier alternatives to common animal fat in the design and development of meat products with reduced fat content and digestibility. Adding Si from DP to GEs can help improve the stability and rheological properties of meat products and may become an important component of healthier meat functional foods. Further research on this topic is being conducted to confirm these findings, focusing on developing gel-emulsion type meat products, such as pâtés, frankfurters, etc. with these GEs.

## 4. Materials and Methods

### 4.1. Materials and Chemicals

Pork lard with a fat content of 99.9% was obtained after a clarification process from Iberian pork fat (local supermarket, Madrid, Spain). SPC with 72% protein, was kindly donated by Lactotecnia S.L. Ingredientes Alimentarios, Barcelona, Spain. A pork rind protein extract (PRP with 90% protein of which 70% is collagen and 10% fat) was obtained from Prosur (Murcia, Spain). к-carrageenan was supplied by Tradissimo, Trades S.A. (Barcelona, Spain). MTG with 50 U/g transglutaminase activity (Activa^®^ EB) was donated by Ajinomoto Foods Europe (Paris, France). The food grade preparation (diatomaceous earth powder (DP); Tierra de Diatomeas^®^) with a SiO_2_ richness of 85%, thus containing 40% Si, was kindly donated by Vitality Gest S.L. (Valencia, Spain). Pepsin (≥2500 U/mg protein, P7012), pancreatin from porcine pancreas (8 x USP, P7545), and bile extract porcine (B8631), Fast Green FCF (F7252), Red Nile (72,485), dehydrate calcium chloride, potassium chloride, sodium bicarbonate, and sodium hydrogen carbonate were supplied by Sigma Aldrich Chemie GmbH (Steinheim, Germany). Sodium chloride, hydrochloric acid 37%, sodium hydroxide pellets, trichloroacetic acid, sulfate ammonium, and hexane were supplied by Panreac (Barcelona, Spain).

### 4.2. Preparation of Gelled Emulsions (GE)

Gelled O/W emulsions (40/60) were prepared in a 2-step emulsification procedure. First, primary emulsions stabilized with SPC or PRP were prepared as described in Cofrades et al. [18] with the following modifications. The oil phase consisted of melted pork lard and the aqueous phase contained the emulsifying protein (SPC or PRP), which was hydrated in part of the water of the aqueous phase. The structuring solutions (MTG and к-carrageenan), prepared with the rest of the water of the aqueous phase, were added to the primary emulsions and mixed using a rod stirrer (Bunsen AGV-8, Madrid, Spain) at 500 rpm/1 min until complete homogenization. These emulsions were labeled and hereafter referred to as SPC (GE1) and PRP (GE2). The same procedure was used to prepare the emulsions with DP, which was added to the melted oil phase and stirred for 30 min just before adding it to the aqueous phase. These emulsions were labeled and hereafter referred to as SPC- DP (GE3) and PRP- DP (GE4). Aliquots of ≈ 10 g were rapidly poured into cylindrical-shaped containers (3.5 cm height × 2.5 cm diameter) with lids and stored at 4 ± 2 °C to form the final GE until analysis after 1, 7, 14, and 28 days of storage. GE1 contained 3% SPC (*w*/*w*); GE2 contained 3% PRP (*w*/*w*); GE3 contained 3% SPC and 1.5% DP (*w*/*w*) (containing 0.6 g Si); and GE4 contained 3% PRP (*w*/*w*) and 1.5% DP (*w*/*w*). All four GEs contained 40% clarified pork fat, 1% MTG (*w*/*w*), and 0.75% к-carrageenan (*w*/*w*) as gelling agents.

### 4.3. Microstructure

The microstructure was determined in freshly prepared or non-digested GEs and after simulated GID phases (gastric digestion; GD, intestinal digestion (ID) at min 5; ID–5, and ID at min 90; ID–90) by confocal laser scanning microscopy (CLSM). Images were collected using a confocal microscope (Leica TCS SP5 AOBS, Mannheim, Germany) with a 20× objective, and some fields of each sample with a 5× zoom as previously described [18]. 

### 4.4. Instrumental Texture and Color

A penetration test was performed in a TA.HDPlus Texture Analyzer (Stable Micro Systems, Ltd., Godalming, UK) equipped with a 5 kg load cell. A 4 mm cylindrical stainless-steel flat probe was used to penetrate the sample placed in the cylindrical-shaped container up to 10 mm at a speed of 1 mm/s. The samples were tempered at 20 °C before the measurements. The penetration force at 10 mm (N) and the penetration work (mJ) were derived from the force-distance curves. Color differences between formulations and during chilling storage were determined by reflectance (Chroma Meter CR 400, Konica Minolta Sensing, Inc., Osaka, Japan) using the CIE Lab scale (D65/10°), where L* (black 0 to light 100), a* (red 60 to green –60) and b* (yellow 60 to blue –60) were used to measure lightness, redness, and yellowness.

### 4.5. Gravitational and Thermal Stability

As a measure of emulsion stability, gravitational and heating losses were determined. Gravitational stability was determined by measuring the exudate released during chilling storage at 4 °C. Samples were removed from the cups and allowed to stand until they reached room temperature; then, they were superficially dried with a paper towel. The exudate released was expressed as a percentage of the initial sample weight. Heating loss was measured to determine the thermal stability (fat and water binding properties) of the GE. The containers with all GE were introduced in a water bath for 30 min at 70 °C, and then opened and left to stand upside down (for 30 min) to release the exudate.The heating loss was expressed as % of the initial sample weight.

### 4.6. Determination of Thiobarbituric Acid Reactive Substances)

TBARS in all GEs were determined according to the method described by Freire et al. [1]. In brief, 0.25 g of each GE was mixed with 200 μL of BHT (1% in distilled water), 550 μL of water, and 2 mL of a TBARS reagent freshly prepared. This mixture was placed in a boiling water bath for 15 min and then cooled in ice. After that, 1 mL of 4 M ammonium sulfate was added to the mixture and vortexed for 1 min, after which 1 mL of hexane was added and vortexed and then centrifuged at room temperature at 662 g for 30 min (Multifuge 3 LR, Heraeus, DJB Labcare Ltd.; Buckinghamshire, UK). Absorbance of the lower phase was measured at 532 nm. A calibration curve was plotted using 1,1,3,3-tetraethoxypropane (Sigma-Aldrich, Madrid, Spain), as the malondialdehyde (MDA) source, and the results were expressed as mg MDA/kg of sample.

### 4.7. In Vitro Gastrointestinal Digestion (GID)

24 h after their formulation and storage at 4 °C, the GEs were subjected to the in vitro INFOGEST 2.0 digestion procedure, based on the protocol described by Brodkorb et al. [31] with slight modifications. The complete simulated in vitro GID procedure, including oral, gastric, and intestinal phases are described briefly:

Oral phase: 2.5 g of each emulsion (containing 1 g of fat) was added to 2.5 mL milli-Q water and then mixed with 4 mL of simulated salivary fluid electrolyte (eSSF) with pH 7.0 and no salivary α-amylase (no starch in the emulsion), 0.025 mL CaCl_2_ (0.3 M), and 0.975 mL of milli-Q water. The bolus was incubated at 37 °C for 2 min under continuous stirring using a mechanical shaking device to mimic oral digestion.

Gastric phase: 8 mL of simulated gastric fluid electrolyte (eSGF) solution was added to the oral bolus (10 mL) to obtain a final ratio of 1:1 (*v*/*v*), followed by 0.005 mL of CaCl_2_ (0.3 M), and the pH was adjusted to 3.0 using HCl 1 N. Then, freshly prepared porcine pepsin prepared in milli-Q water was added to achieve an activity of 2000 U/mL in the final digestion mixture. The mixture was incubated at 37 °C for 60 min under continuous agitation using the above-mentioned mechanical shaking device. During the time of gastric digestion, the pH was adjusted to 3.0 as required. Finally, the necessary amount of milli-Q water was added to obtain 10 mL of simulated gastric fluid.

Intestinal phase: 7 mL of electrolyte simulated intestinal fluid (eSIF) and 0.04 mL of CaCl_2_ (0.3 M) were added to the chyme (20 mL), and the pH was adjusted to 7.0 with NaOH 1 N under continuous agitation at 37 °C. A solution of bile extract (10 mM in 4 mL eSIF) was then added and, if necessary, the pH was readjusted to 7.0. Thereafter, freshly prepared porcine pancreatin in eSIF was added to achieve a lipase activity of 2000 U/mL in the final mixture. An automatic titration device (TitroMatic 1 S, Crison, Alella, Spain) was used to maintain the sample at pH 7.0 by adding NaOH (1 N). The sample was kept under simulated small intestinal conditions for 90 min. To inhibit lipolysis, 200 µL of 5 mM 4-bromophenylboronic acid were added to the final digested mixture and stored at −20 °C until used.

### 4.8. Fat Extraction, High-Performance Size-Exclusion Liquid Chromatography, and Extent of Lipolysis during In Vitro GID

The lipid composition of TAG, DAG, MAG, and FFA was determined in pork lard and in the 4 emulsions after 90 min of intestinal digestion (ID–90) as described previously. 

The digestibility of the samples was calculated according to the following formula:

*Digestibility* (%) = [(*TAG_initial_* − *TAG_t_*)/*TAG_initial_*] × 100

Here, *TAG_initial_* is the proportion of TAG in the fat present in all the non-digested GEs and *TAG_t_* is the proportion of TAG in the fat of the digestion product in ID–90.

### 4.9. Statistical Analysis

For the initial studies (day 1), all the GEs were prepared in triplicate. Then, from each replicate, the analyses (texture, color, binding properties, and in vitro GID) were performed in triplicate. For all measurements, except for the fat composition after in vitro digestion, the effect of GE composition (emulsifying protein (EP) and Si presence or absence) for the same chilling storage time was studied by a one-way analysis of variance (ANOVA). The difference in means between pairs was determined using confidence intervals with the Bonferroni or Tamhane tests, depending on the variance homogeneity. In the case of the fat composition, the following comparisons were performed using *t*-tests: GE1 vs. GE3; GE2 vs. GE4; GE1 vs. GE2; and GE3 vs. GE4. In addition, for all measurements except for the fat composition after in vitro digestion, a mixed model analysis of ANOVA with repeated measures during time was used to analyze the effect of chilling storage time for each GE. The difference of means between pairs was resolved using confidence intervals with the Bonferroni test. In all analyses, the letters “a” and “A” were assigned the highest values. The level of significance was set at *p* ≤ 0.05. The statistical analysis was performed using the SPSS statistical program (version 27.0, SPSS Inc., Chicago, IL, USA). Results are expressed as mean ± standard deviation.

## Figures and Tables

**Table 1:** Instrumental color parameters for lightness (L*), redness (a*), and yellowness (b*) of the GEs over chilling storage time.

Gelled Emulsion	Number of Days	L*	a*	b*
GE1	1	88.08 ± 0.53 ^A^_a_	−0.21 ± 0.04 ^B^_b_	5.94 ± 0.07 ^B^_a_
GE2	1	88.25 ± 0.11 ^A^_a_	−0.55 ± 0.08 ^A^_b_	5.61 ± 0.13 ^C^_a_
GE3	1	86.50 ± 0.66 ^B^_ab_	0.05 ± 0.04 ^C^_a_	6.28 ± 0.05 ^A^_a_
GE4	1	88.37 ± 0.57 ^A^_a_	−0.27 ± 0.02 ^B^_ab_	5.48 ± 0.11 ^C^_a_
GE1	7	88.24 ± 0.80 ^AB^_a_	−0.10 ± 0.13 ^B^_b_	5.48 ± 0.17 ^AB^_b_
GE2	7	88.28 ± 0.42 ^AB^_a_	−0.39 ± 0.08 ^A^_b_	5.25 ± 0.06 ^B^_b_
GE3	7	87.41 ± 0.28 ^B^_a_	0.14 ± 0.05 ^C^_a_	5.88 ± 0.01 ^A^_bc_
GE4	7	88.79 ± 0.64 ^A^_a_	−0.07 ± 0.11 ^BC^_b_	5.15 ± 0.19 ^B^_a_
GE1	14	87.98 ± 0.46 ^A^_a_	−0.05 ± 0.11 ^C^_b_	5.71 ± 0.29 ^A^_ab_
GE2	14	88.38 ± 0.30 ^A^_a_	−0.41 ± 0.03 ^A^_b_	5.35 ± 0.12 ^AB^_a_
GE3	14	86.56 ± 0.18 ^B^_b_	0.33 ± 0.07 ^B^_a_	5.62 ± 0.17 ^AB^_c_
GE4	14	87.79 ± 0.48 ^A^_a_	0.14 ± 0.18 ^BC^_b_	5.21 ± 0.13 ^B^_a_
GE1	28	87.66 ± 0.20 ^AB^_a_	−0.61 ± 0.10 ^B^_a_	5.85 ± 0.07 ^AB^_ab_
GE2	28	88.09 ± 0.50 ^A^_a_	−0.90 ± 0.07 ^A^_a_	5.67 ± 0.18 ^B^_a_
GE3	28	86.90 ± 0.60 ^B^_ab_	−0.30 ± 0.04 ^C^_b_	6.10 ± 0.10 ^A^_ab_
GE4	28	87.99 ± 0.53 ^A^_a_	−0.47 ± 0.10 ^BC^_a_	5.34 ± 0.15 ^C^_a_

Mean values (*n* = 4) ± standard deviation. ^A–C^ Effect of GE composition (emulsifying protein (EP) and silicon (Si) presence). For each color parameter and for the same chilling storage time, mean values without the same letter are significantly different (*p* < 0.05). _a–c_ Effect of chilling storage. For each color parameter and for the same GE, mean values without the same letter are significantly different (*p* < 0.05). GE, gelled emulsion; GE1, containing 3% soy protein concentrate (SPC); GE2, containing 3% pork rind protein extract (PRP); GE3, containing 3% SPC and 1.5% diatomaceous powder (DP); and GE4 containing 3% PRP and 1.5% DP. All four GEs contained 40% pork fat, 1% MTG, and 0.75% к-carrageenan.

**Table 2:** Fat composition (g/100 g fat) and % of digestibility of gelled emulsions after in vitro GID performed in a pH stat after 90 min intestinal digestion.

Gelled Emulsion	TAG	DAG	MAG	FFA	MAG + FFA	Digestibility
Initial pork lard	99.9 ± 0.0					
GE1	10.41 ± 1.91 ^B^_b_	11.47 ± 0.49 ^A^_b_	15.96 ± 1.95 ^A^_a_	62.16 ± 2.69 ^A^_a_	78.12 ± 1.62 ^A^_a_	89.58 ± 1.91 ^A^_a_
GE2	18.12 ± 1.64 ^A^_b_	11.42 ± 0.85 ^A^_b_	15.27 ± 0.22 ^A^_a_	55.20 ± 0.93 ^B^_a_	70.47 ± 1.07 ^B^_a_	81.45 ± 1.77 ^B^_a_
GE3	14.84 ± 1.67 ^B^_a_	12.32 ± 0.70 ^A^_a_	14.53 ± 0.54 ^A^_b_	58.31 ± 0.81 ^A^_b_	72.84 ± 1.10 ^A^_b_	85.14 ± 1.68 ^A^_b_
GE4	21.99 ± 3.89 ^A^_a_	13.15 ± 0.54 ^A^_a_	12.51 ± 1.19 ^A^_b_	52.35 ± 2.98 ^B^_b_	64.86 ± 4.17 ^B^_b_	77.99 ± 3.90 ^B^_b_

Mean values (*n* = 3) ± standard deviation. TAG, triacylglycerides; DAG, diacylglycerides; MAG, monoacylglycerides; FFA: Free Fatty Acids; MAG, monoacylglycerides; GE, gelled emulsion. ^A,B^ Effect of the emulsifying protein (EP) in GEs. For the same Si level (presence or absence), mean values without the same letter are significantly different (*p* < 0.05). _a,b_ Effect of silicon (Si) presence in GEs. For the same emulsifying protein (SPC or PRP), mean values without the same letter are significantly different (*p* < 0.05). For sample denomination, see Table 1.

## Data Availability

Data will be made available on request.
